# Associations of plasma C-Reactive Protein and osteopontin levels with the severities of coronary and aortic atherosclerosis

**DOI:** 10.1186/1532-429X-11-S1-P208

**Published:** 2009-01-28

**Authors:** Yukihiko Momiyama, Reiko Ohmori, Zahi A Fayad, Teruyoshi Kihara, Ryuichi Kato, Hiroaki Taniguchi, Masayoshi Nagata, Haruo Nakamura, Fumitaka Ohsuzu

**Affiliations:** 1 National Hospital Organization Tokyo Medical Center, Tokyo, Japan; 2 National Defense Medical College, Saitama, Japan; 3 Mount Sinai School of Medicine, New York, NY USA; 4 Iruma Heart Hospital, Saitama, Japan

**Keywords:** Abdominal Aorta, Plaque Score, Aortic Atherosclerosis, Stenotic Segment, Stenotic Vessel

## Introduction

Limited correlations between biomarkers (e.g. C-reactive protein (CRP) and osteopontin (OPN)) and the severity of coronary artery disease (CAD) have been reported. Recently, MRI became a useful tool for non-invasively evaluating atherosclerotic plaques in both thoracic and abdominal aortas. Using MRI, we investigated the associations of plasma CRP and OPN levels with the severities of coronary and aortic atherosclerosis.

## Methods

Aortic MRI was performed on Signa 1.5 T Cvi with a phased-array body coil in 136 patients undergoing coronary angiography. Transverse PDW and T2W images of thoracic descending and abdominal aortas were obtained using an ECG-gated, double-inversion-recovery FSE sequence. Imaging parameters were TR = 2 RR intervals, TE = 10 (PDW) and 60 ms (T2W), 20-cm FOV, 4-mm slice thickness, 8-mm inter-slice gap, 256 × 256 acquisition matrix, and 32 echo-train. For each patient, 9 slices of thoracic aorta and 9 slices of abdominal aorta were obtained at 12-mm intervals, which each covered about 10-cm portion of thoracic aorta below the arch and 10-cm portion of abdominal aorta above the bifurcation of iliac artery. Plaque was defined as a clearly identified luminal protrusion with focal wall thickening, and plaque extent in each slice was scored 0 to 4 points by the percentage of luminal surface involved by plaque. The severity of aortic atherosclerosis was represented as the sum of scores (plaque score). On coronary angiograms, the degree of stenosis in each segment was evaluated by 5 grades. The severity of coronary atherosclerosis was represented as the number of >50% stenotic vessels and the number of >25% stenotic segments. Plasma OPN and high-sensitivity CRP levels were measured by ELISA with a commercially available kit (Human OPN assay kit, IBL) and by a BNII nephelometer (Dade Behring), respectively. Any patients with acute coronary syndromes were excluded from this study.

## Results

Of the 136 patients, 53 (39%) were on statin treatment, and 96 (71%) had CAD (>50% luminal diameter stenosis) on angiograms. Thoracic and abdominal aortic plaques were found in 88 (65%) and 124 (91%) patients, respectively. Patients with CAD had higher OPN (562 ± 223 vs. 445 ± 234 ng/ml) and CRP (median 0.78 vs. 0.48 mg/l) levels than those without CAD (P < 0.02). Plasma OPN levels did not correlate with CRP levels. Stepwise increase in OPN levels was found depending on the number of >50% stenotic coronary vessels: 445 ± 234 (0VD), 541 ± 239 (1VD), 556 ± 219 (2VD), and 604 ± 211 ng/ml (3VD) (P < 0.05). OPN levels correlated with the number of >25% stenotic coronary segments (r = 0.23 by Spearman's rank correlation test, P < 0.01). Plasma CRP levels also stepwise increased depending on the number of stenotic vessels: 0.48, 0.70, 0.74, and 0.88 mg/l (P = NS). CRP levels correlated with the number of stenotic segments (r = 0.21 by Spearman's rank correlation test, P < 0.02). Regarding aortic atherosclerosis, the 136 patients were divided into quartiles by plaque score. Stepwise increase in OPN levels was found depending on the quartiles of aortic plaque score: 451 ± 165 (Q1), 471 ± 236 (Q2), 560 ± 232 (Q3), and 629 ± 252 ng/ml (Q4) (P < 0.02). OPN levels correlated with aortic plaque score (r = 0.26, P < 0.005). CRP levels also stepwise increased on the quartiles: 0.40, 0.56, 1.08, and 1.10 mg/l (P < 0.001). CRP levels correlated with plaque score (r = 0.38, P < 0.001). In multivariate analysis, aortic atherosclerosis was found to be an independent factor associated with both OPN and CRP levels, but coronary atherosclerosis was not.

## Conclusion

Plasma OPN and CRP levels correlated with the severities of both coronary and aortic atherosclerosis, but these biomarkers are more likely to reflect the severity of aortic atherosclerosis than coronary atherosclerosis. MRI was useful for non-invasively evaluating atherosclerosis in thoracic and abdominal aortas.

